# Sequence‐based searching of custom proteome and transcriptome databases

**DOI:** 10.14814/phy2.13846

**Published:** 2018-09-19

**Authors:** Barbara Medvar, Abhijit Sarkar, Mark Knepper, Trairak Pisitkun

**Affiliations:** ^1^ Epithelial Systems Biology Laboratory Systems Biology Center National Heart, Lung, and Blood Institute National Institutes of Health Bethesda Maryland; ^2^ Vitreous State Laboratory The Catholic University of America Washington District of Columbia; ^3^ Physics Department The Catholic University of America Washington District of Columbia; ^4^ Center of Excellence in Systems Biology Faculty of Medicine Chulalongkorn University Bangkok Thailand

**Keywords:** BLAST, database, IMCD, kidney

## Abstract

A long‐term goal in renal physiology is to understand the mechanisms involved in collecting duct function and regulation at a cellular and molecular level. The first step in modeling of these mechanisms, which can provide a guide to experimentation, is the generation of a list of model components. We have curated a list of proteins expressed in the rat renal inner medullary collecting duct (IMCD) from proteomic data from 18 different publications. The database has been posted as a public resource at https://hpcwebapps.cit.nih.gov/ ESBL/Database/IMCD_Proteome_Database/. It includes 8956 different proteins. To search the IMCD Proteomic Database efficiently, we have created a Java‐based program called *curated database Basic Local Alignment Search Tool* (cdbBLAST), which uses the NCBI BLAST kernel to search for specific amino acid sequences corresponding to proteins in the database. cdbBLAST reports information on the matched protein and identifies proteins in the database that have similar sequences. We have also adapted cdbBLAST to interrogate our previously published IMCD Transcriptome Database. We have made the cdbBLAST program available for use either as a web application or a downloadable .jar file at https://hpcwebapps.cit.nih.gov/ ESBL/Database/cdbBLAST/. Database searching based on protein sequence removes ambiguities arising from the standard search method based on official gene symbols and allows the user efficient identification of related proteins that may fulfill the same functional roles.

## Introduction

With the advent of large‐scale proteomic and transcriptomic experiments for profiling gene expression, data access and integration has become rate‐limiting for acquisition of biological knowledge. Access is facilitated through the creation of databases of curated datasets. For example, our laboratory alone has generated approximately 70 such databases, accessible at https://hpcwebapps.cit.nih.gov/ESBL/Database/index.html. Because of the large amount of data within these types of databases, it becomes difficult to find information about a specific protein/transcript. The question then becomes: what is the best way to find a particular protein/transcript within the databases? A general strategy to database searches is to utilize so‐called “keys” or “indices” that constitute finite search target lists. In systems biology, there are a few common “keys,” namely a common name for a protein/transcript, a protein/transcript's gene symbol, or the amino acid/nucleotide sequence for a protein/transcript. Problems arise when a database is searched using a common name for the protein/transcript in question; common or large proteins have multiple names and can be difficult to find if the user is not searching for the name used within the database. Searching using a gene symbol is a better option, however it is not without its own difficulties. As with searching using a common name, a well‐studied protein can be linked to multiple gene symbols. A second issue that arises from using the gene symbol as the “key” to searching databases is the inability to comprehensively search for related proteins. Unless all proteins share a similar gene symbol (e.g., Aqp1, Aqp2, etc.), it is extremely difficult to find all related proteins, for example, those with a particular domain. The best solution to the problems that arise from searching by gene symbol or a common name is to search curated databases using a protein/transcript's amino acid/nucleotide sequence. Each protein/transcript has a unique sequence, allowing the correct protein/transcript to be found regardless of the gene symbol or name used in the database. Using a sequence also allows for related proteins/transcripts to be found based on similar sequence matches.

The primary goal of this paper is to introduce a sequence‐based search tool, called cdbBLAST (curated database BLAST), that uses NCBI's *blastp* kernel to uniquely find a protein/transcript within a curated database, as well as find similar proteins/transcripts, and provides information from the database on each of the proteins/transcripts in the results. Another benefit of cdbBLAST is that it allows for domain sequence searches, so it is possible to find all proteins/transcripts within a curated database that share a domain. We illustrate the use of this tool by applying it to two newly curated databases, a database of all rat inner medullary collecting duct transcripts, and a database of all rat inner medullary collecting duct proteins. The former is created by combining two published studies, one from RNA‐seq and one from expression microarrays, while the latter is a created by distilling information from 18 published studies.

## Methods

We curated a comprehensive list of proteins found in the rat IMCD cells from 18 studies (van Balkom et al. 2004; Hoffert et al. 2004, 2006, 2012, 2014; Barile et al. 2005; Hoorn et al. 2005; Pisitkun et al. 2006; Simons et al. 2006; Yu et al. 2006; Sachs et al. 2008; Bansal et al. 2010; Tchapyjnikov et al. 2010; Zhao et al. 2012; Bradford et al. 2014; Trepiccione et al. 2014; Pickering et al. 2016; LeMaire et al. 2017) done within our laboratory. Some of these proteomic studies include multiple experimental methods, which results in 20 “sources” describing which experimental method identified a specific protein. This database has been put together on a publicly accessible webpage ( https://hpcwebapps.cit.nih.gov/ESBL/Database/IMCD_Proteome_Database/).

We have created a sequence‐based search tool, called cdbBLAST, which allows databases to be searched for a certain protein using a protein‐to‐protein sequence comparison. cdbBLAST uses the NCBI *blastp* kernel to search the database for the protein in question and returns similar proteins that are found in the database as well. It also returns information from the database on each of the proteins in the results. cdbBLAST can be used as a web‐based servlet or as a downloadable GUI.

**Figure 1:**
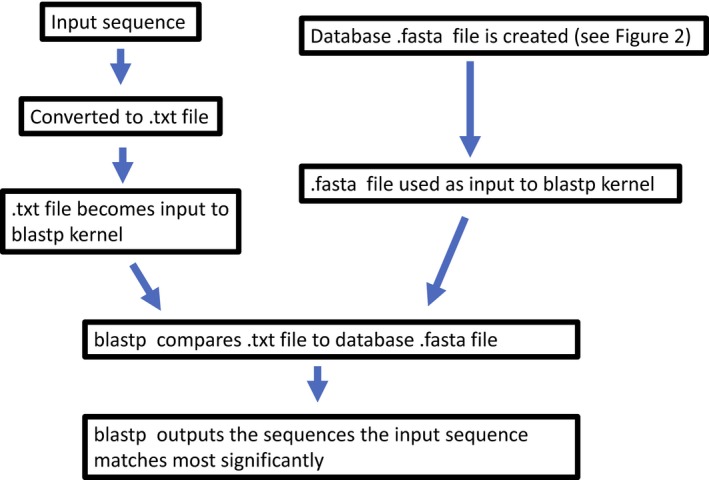
A flowchart explaining the algorithm of cdbBLAST. The input sequence is converted to a .txt file, which becomes the input to the BLAST kernel. This input file is compared to a .fasta database, and the sequences in the .fasta database that match most closely to the input sequence are output in the results.

When the user types (or pastes) a string of amino acids into the search box and hits submit, cdbBLAST turns that string into a .txt file, and uses it as input to the NCBI *blastp* kernel. The input needs to be a minimum of 11 amino acids in length. The blastp kernel then compares this input file to a database of sequences, and prints the results of the comparison (Fig. 1). The cdbBLAST web‐based servlet then prints these results as a webpage. The cdbBLAST GUI version outputs the results directly in blastp format.

### Software implementation

cdbBLAST, both the web‐based servlets and the downloadable version, were written in Java (Java Development Kit 1.8 Update 121) using NetBeans IDE 8.1 as an integrated development environment. They were implemented using Apache Tomcat 8.0.15. The 2.4.0 version of *blastp* kernel from NCBI is used, and was downloaded from ftp://ftp.ncbi.nlm.nih.gov/blast/executables/blast+ as part of the blast 2.4.0+ folder.

cdbBLAST takes an amino acid sequence as input and compares it to a list of FASTA sequences corresponding to entries in the curated database. This list includes not only FASTA sequences and the typical FASTA metadata, but additional information from the databases that are built into the metadata.

**Figure 2:**
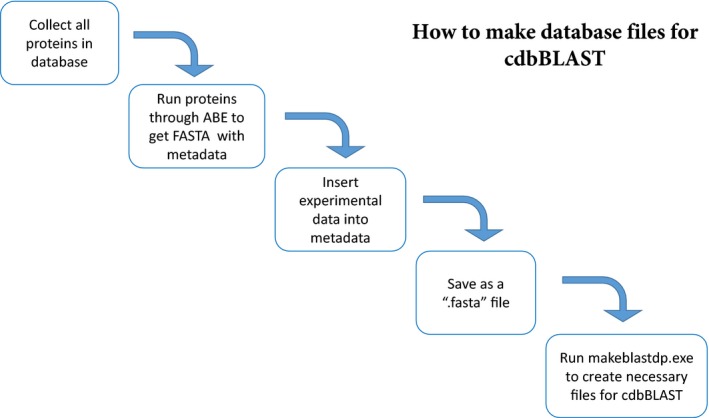
A flowchart explaining the process for creating a .fasta database to be used with cdbBLAST. We start with a list of all proteins* in the database. That list is then run through Automated Bioinformatics Extractor (ABE) to get the FASTA sequence and metadata associated with each protein in the list. Next, experimental information is added to the metadata for each protein, and this new list is saved as a “.fasta” file. The .fasta file is then the input for makeblastdb.exe, which creates the three necessary files needed to run cdbBLAST. A more detailed explanation can be found in the Appendix, downloadable from https://hpcwebapps.cit.nih.gov/ ESBL/Database/cdbBLAST‐Appendix/Appendix.html. *This figure describes proteins being made into a database. The process for creating a database of transcripts is the same.

These FASTA sequence lists were created using the Automated Bioinformatics Extractor (ABE) to convert the *RefSeq* protein accession numbers of the entries to FASTA amino acid sequence and metadata. The database information was then combined with the existing metadata for each protein/transcript. Next the list was copied into a Notepad file, and saved as a “filename.fasta” file. Subsequently, in the command line, the BLAST code makeblastdb.exe was run, using this new .fasta file as part of the input. This results in three new files being created: filename.fasta.phr, filename.fasta.pin, and filename.fasta.psq. These three files are required to run blastp, and thus cdbBLAST (Fig. 2). A more thorough step by step process can be found in the Appendix, downloadable from https://hpcwebapps.cit.nih.gov/ESBL/Database/cdbBLAST-Appendix/Appendix.html.

## Results

**Figure 3:**
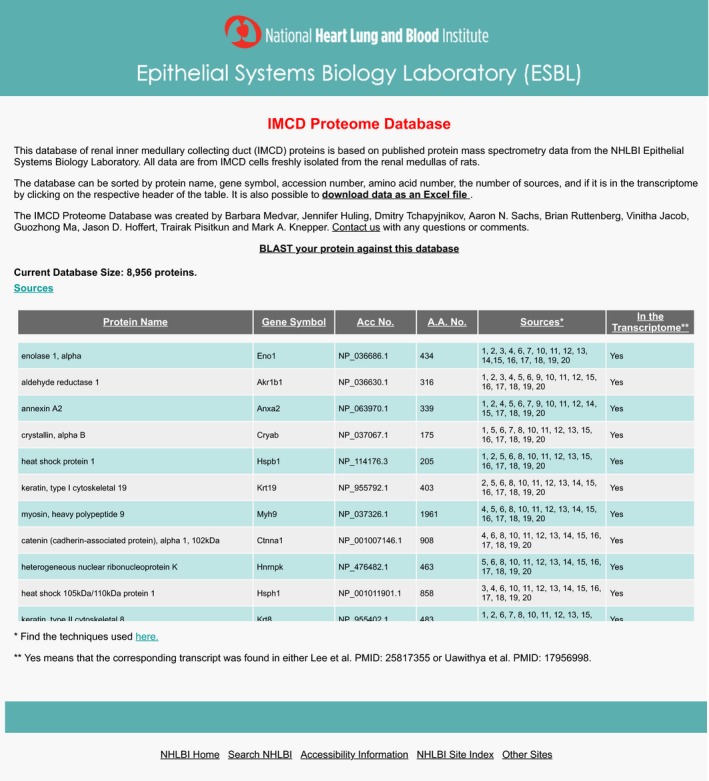
A publicly available webpage was created to provide users with a complete list of proteins found in the rat IMCD, as identified in 18 studies. Access to this webpage can be found at https://hpcwebapps.cit.nih.gov/ESBL/Database/IMCD_Proteome_Database/. There is a link to a list of all sources, as well as a link to download the data to an Excel file. The data on this webpage can be sorted by the number of sources a protein is found in, amino acid number, if a protein is found in the transcriptome, or alphabetically by gene symbol, protein name, or RefSeq number.

We have curated a rat IMCD proteome database from 18 studies produced in our laboratory, resulting in a database of 8956 proteins. This database has been made into a publicly accessible webpage ( https://hpcwebapps.cit.nih.gov/ESBL/Database/IMCD_Proteome_Database/) (Fig. 3). This database shows how many different experimental datasets each protein was found in, as well as its protein name, official gene symbol, accession number, and number of amino acids. The database also shows, and can be sorted by, whether the corresponding mRNA is present in the transcriptome.

**Figure 4:**
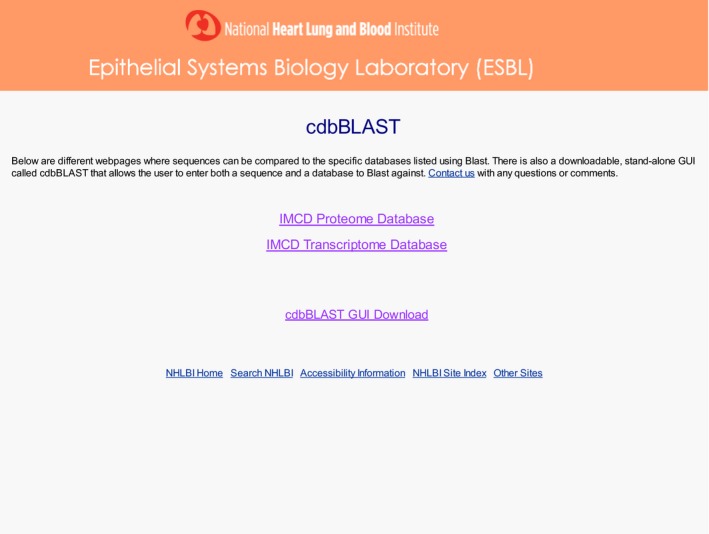
A screenshot of the main cdbBLAST webpage. This page has links to the IMCD Proteome and Transcriptome cdbBLAST searches, which compare an input sequence to our databases of proteins and transcripts, respectively. There is also a link to the downloadable GUI version of cdbBLAST, where the user can compare a sequence of interest to their own database of sequences.

To best search large databases like this rat IMCD proteome, we have created a sequence‐based search tool called cdbBLAST. cdbBLAST allows the user to input a full amino acid sequence, or a partial sequence, and compare it against a list of FASTA sequences corresponding to the proteins/transcripts in the curated database. We have created a rat IMCD proteome database and combined two existing rat IMCD transcriptome databases to be searched using cdbBLAST, which has been made publicly accessible (https://hpcwebapps.cit.nih.gov/ESBL/Database/cdbBLAST) as well as downloadable as a .jar file (Fig. 4).

**Figure 5:**
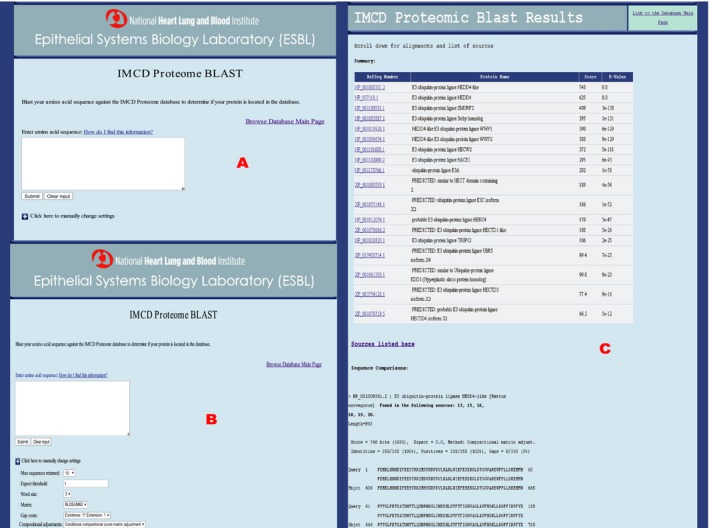
A screenshot of the rat IMCD Proteomic cdbBLAST (A) initial page where a sequence can be typed or pasted into the white box, (B) settings that can be changed to alter the results of a search, and (C) results, using Nedd4l as a sample amino acid input sequence. The results include a summary table of the proteins whose sequences best match the query (input) sequence, which includes links to their PubMed protein pages. Below this table are comparisons of these top proteins to the query sequence. Within each comparison, the sources in which that top protein is found are listed. There is a link to the original database on both the initial page and the results page.

The proteome cdbBLAST search (Fig. 5A) allows the user to input an amino acid sequence and search the curated rat IMCD proteome database introduced in this paper. There is a link on both the main page and the results page of cdbBLAST to the proteome database. At the bottom of the search page, there is a set of criteria (e.g., expect threshold, word size, substitution matrix, and gap costs) the user can use to manipulate the results, based on NCBI's *BLAST* code (Altschul et al. 1990) (Fig. 5B). Once the user is content with the criteria and the sequence used, hitting “submit” will allow the code to run and brings up a results page. This results page shows a table at the top of the page with the proteins that match best to the search sequence. It also shows the related proteins’ RefSeq number, which is a link to that protein in PubMed (National Center for Biotechnology Information, 2017), and the “score” and “e‐value” as determined by NCBI's *blastp* code. The “score” is a normalized score for aligning pairs of residues between the query and the database sequences, where the “e‐value” or expected value is the number of hits one can expect to see by chance. The higher the score, and the lower the e‐value, the more significant the match. Below this table, each related protein is compared to the search sequence. Each comparison is labeled with the protein's RefSeq number, protein name, and what sources from the IMCD proteome that protein was found in. Between the table and the comparisons is a link labeled “Sources listed here” which allows the user to open the “Sources” page from the IMCD proteome database in another tab so they are able to see what each numbered source is without losing their cdbBLAST search results. To show an example of what the results look like, we used the partial sequence associated with the HECTc domain from Nedd4l as a sample query. Nedd4l is an E3 ubiquitin ligase most likely to ubiquitinate the water channel aquaporin‐2 (Medvar et al. 2016) in IMCD cells. One use of cdbBLAST is searching a large‐scale database for an amino acid sequence associated with a specific domain to find all proteins within that domain. cdbBLAST is a useful tool for this type of search because of its ability to give not only results that match exactly, but also results that are similar. As seen in Figure 5C, using the HECTc domain of Nedd4l shows results not only for Nedd4l, but also for 17 similar proteins. For Nedd4l you can also see the beginning of the sequence comparison, as well as the datasets Nedd4l was found in.

**Figure 6:**
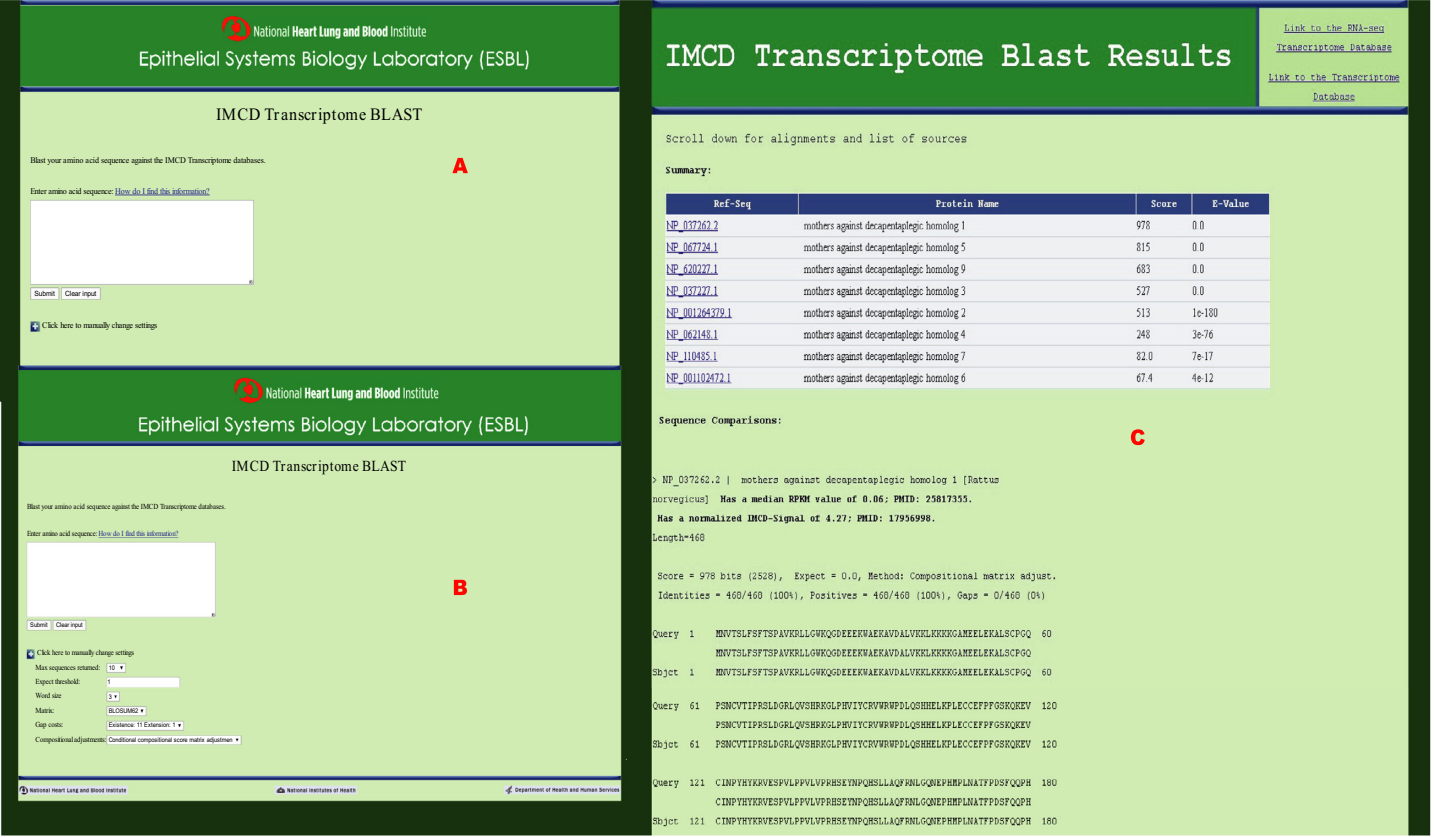
A screenshot of the rat IMCD Transcriptome cdbBLAST (A) initial page where a sequence can be typed or pasted into the white box, (B) settings that can be changed to alter the results of a search, and (C) results, using Smad1 as a sample amino acid input sequence. There are links to the original databases, a summary table of the transcripts whose sequences best match the query (input) sequence which includes links to their PubMed protein pages, and comparisons of these top transcripts to the query sequence. Each comparison also reports the median RPKM value and/or the normalized IMCD‐Signal (depending what study that protein is found in) for that transcript.

The rat IMCD transcriptome cdbBLAST search (Fig. 6A) is very similar to the proteome search. However, this database is a combination of two different transcriptomic databases: one using RNA‐sequencing (Lee et al. 2015) and the other using Affymetrix techniques (Uawithya et al. 2008). The results from this cdbBLAST search are structured the same as the proteome cdbBLAST search (see above), however the comparisons are labeled with the protein's RefSeq number, protein name, and the median RPKM value and/or the normalized IMCD‐Signal (depending what study that protein is found in). As an example, we searched the IMCD transcriptome using the amino acid sequence for Smad1. Smad1 is a transcription factor found in the IMCD transcriptome as seen in Figure 6C. cdbBLAST also found many very similar proteins as well, some so similar they have the same e‐value as Smad1. The comparison of the query to the database shows that Smad1 was found in both transcriptomic studies used to create this database.

**Figure 7:**
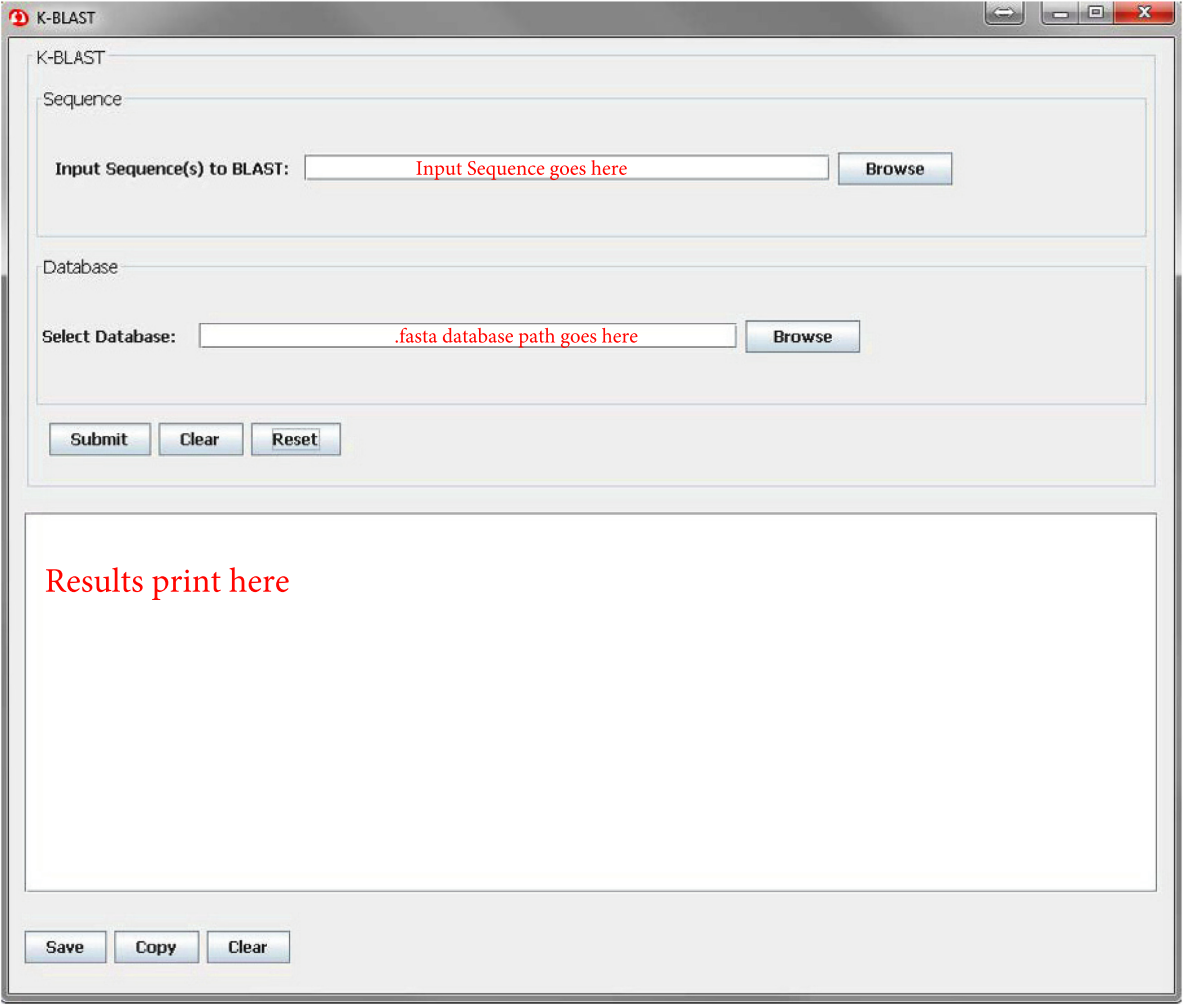
A screenshot of the downloadable version of cdbBLAST. Users can either type/paste in a sequence into the “Input Sequence to BLAST:” box or browse their computer for a .txt file to input. Users then browse their computer (or type in the path) for the .fasta database they either created (Fig. 2) or downloaded from our webpage before hitting “Submit”. “Clear” clears only the input, while “Reset” clears the database and output sections as well. The results print similarly to the web‐servlets, without any links to webpages. The results can be saved, copied, or cleared by hitting the corresponding buttons under the results box.

The downloadable version of cdbBLAST (Fig. 7) is a slightly simpler version than the web servlets. It does not allow the user to adjust the settings and there are no clickable links in the results, but the information provided is the same as the cdbBLAST web‐based searches, with there being a table of similar proteins followed by sequence to sequence comparisons with database data. However, unlike the web servlets, the downloadable version of cdbBLAST can be used with any database of proteins or transcripts that has been correctly formatted. This allows users to integrate any prior data they wish into the database (and thus the output from the search), as well as allows the user to submit multiple sequences as input in a text file. A manual has been created to provide the user with a step‐by‐step guide to this process (see Appendix, download from https://hpcwebapps.cit.nih.gov/ESBL/Database/cdbBLAST-Appendix/Appendix.html). While this is the best use of the cdbBLAST GUI, we have also made the databases created in our laboratory available for download as well on the GUI download page.

## Discussion

Our laboratory has created rat IMCD cdbBLAST searches for the proteome and transcriptome both as web‐based servlets. We have also created a downloadable GUI version, with downloadable versions of our IMCD proteome and transcriptome databases available, as well as a manual explaining how the user can create a database from their own data.

cdbBLAST is a valuable tool that allows scientists to use the most unique search parameter when looking through a database: the amino acid/nucleotide sequence. With large databases created from large‐scale proteomic and transcriptomic experiments becoming more common, a lot of useful data is being reported but not utilized due to the sheer volume of information available. As more is learned about the nature and function of specific proteins or transcripts, they are given new names or gene symbols. This makes older databases tedious and time consuming to search if they cannot be searched via amino acid/nucleotide sequences. There is no longer a need to do multiple searches within a database using every gene symbol from discovery to present in hopes of finding data on a specific protein/transcript.

cdbBLAST also allows the user to search for pieces of sequence, such as protein domains, allowing researchers to learn more about a group of proteins/transcripts in a single search. However, cdbBLAST requires at least 11 amino acids/nucleotides for the search to run, making it difficult search for smaller domains or motifs.

For every searched sequence using cdbBLAST, the results include proteins/transcripts that are similar to the search sequence. This makes it easier for researchers to find a related protein/transcript if the search protein/transcript is not found, or when the searched sequence is a different species than that of the database. Every protein/transcript shown in the results is accompanied by the database information associated with it, as well as a sequence comparison to the searched sequence, so the user can see how well the sequences match. For example, we have combined 18 published studies from our laboratory into one large IMCD proteome database of 8956 proteins. This database lists each proteins name, gene symbol, amino acid number, accession number, whether it is found in the transcriptome, and which of the sources (experiments from within these studies) that protein was found in. When this database is searched using cdbBLAST, each protein in the results lists the sources in which that protein was found. It is logical that the more times a protein was identified, the more likely it is to exist in the IMCD. This added information to the results of the search allows the user to see the relevant information without having to go back to the original database.

The web‐based servlets we have created using cdbBLAST focus on the rat IMCD transcriptome and proteome. Specifically, they focus on the level of expression of the transcripts in the IMCD and what proteins are found in IMCD cells, respectively. The downloadable version of cdbBLAST allows the user to create their own searchable database(s) which can include any and all information they wish, from multiple datasets. Our transcriptome and proteome cdbBLAST databases are also available to be downloaded and used with this version. While amino acid/nucleotide sequences remain consistent through time, more information is learned about proteins and transcripts constantly. With the downloadable version of cdbBLAST, new information can be added to an existing searchable database with ease (see Appendix, download from https://hpcwebapps.cit.nih.gov/ESBL/Database/cdbBLAST‐Appendix/Appendix.html).

## Conflict of Interest

None declared.
